# Wandering Spleen and Organoaxial Gastric Volvulus after Morgagni Hernia Repair: A Case Report and Review of the Literature

**DOI:** 10.1155/2016/6450765

**Published:** 2016-09-14

**Authors:** Noemi Cantone, Caterina Gulia, Vittorio Miele, Margherita Trinci, Vito Briganti

**Affiliations:** ^1^Department of Paediatric Surgery, Spedali Civili Children's Hospital, Brescia, Italy; ^2^Department of Urology, Policlinico Umberto I, Sapienza University, Rome, Italy; ^3^Department of Emergency Radiology, S. Camillo-Forlanini Hospital, Rome, Italy; ^4^Department of Paediatric Surgery, S. Camillo-Forlanini Hospital, Rome, Italy

## Abstract

Wandering spleen and gastric volvulus are two rare entities that have been described in association with congenital diaphragmatic hernia. The diagnosis is difficult and any delay can result in ischemia and necrosis of both organs. We present a case of a 13-year-old girl, previously operated on for anterior diaphragmatic hernia and intrathoracic gastric volvulus, that presented to our service for a subdiaphragmatic gastric volvulus recurrence associated with a wandering spleen. In this report we reviewed the literature, analyzing the clinical presentation, diagnostic assessment, and treatment options of both conditions, in particular in the case associated with diaphragmatic hernia.

## 1. Introduction

Wandering spleen and gastric volvulus are two rare entities that have been described in both adults and children in association with congenital diaphragmatic hernia [1, 2]. The lack of development or incomplete formation of intraperitoneal suspensory ligaments, congenital or secondary to diaphragmatic defect, is a recognized common cause of these conditions [3–5]. The excessive mobility and abnormal position of both stomach and spleen predispose to torsion of these organs that may result in ischemia and necrosis [6–8]. We present a case of a 13-year-old girl previously operated on for right anterior diaphragmatic hernia with gastric volvulus at age 5. Eight years later she returned to our attention because of an acute episode of severe epigastric pain, abdominal distension, and vomiting, after recurrent episodes of vomiting and mild abdominal distension. An abdominal and chest X-ray, abdominal Doppler ultrasound, and abdominal CT with oral contrast permitted the diagnosis of wandering spleen and subdiaphragmatic organoaxial gastric volvulus relapse.

## 2. Case Report

A 13-year-old female presented to our service with an acute episode of severe epigastric pain, abdominal distension, and uncontrolled vomiting. Eight years before, she underwent surgery for a right Morgagni hernia finding an organoaxial gastric volvulus; in that occasion, a primary closure of the diaphragm defect and an anterior Boerema's gastropexy were performed. The postoperative period was characterized by intermittent episodes of epigastric pain, vomiting, and mild upper abdominal distension, which improved spontaneously or with nasogastric tube insertion. Six months before, she was admitted to our hospital for acute gastric distension and retching, which resolved with a nasogastric probe for 48 hours. Oral contrast X-ray did not showed a gastric volvulus.

**Figure 1:**
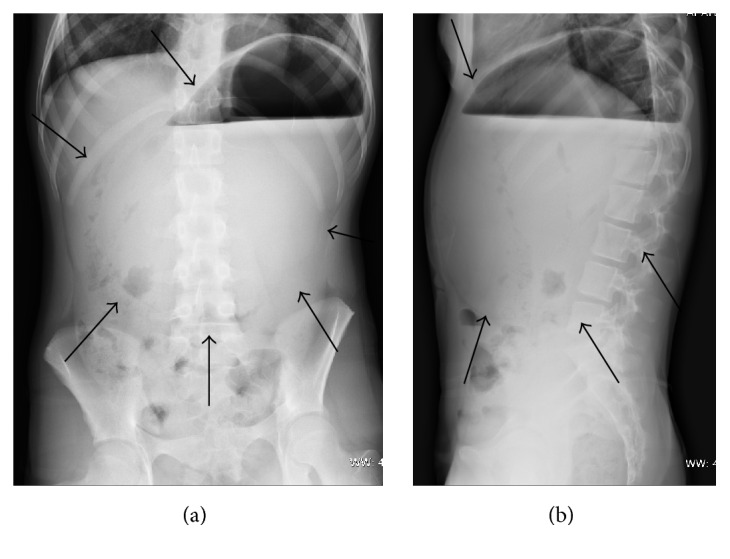
Anteroposterior (a) and lateral (b) projections of the abdominal X-ray. The distended stomach (black arrows) occupies epigastrium and mesogastrium; air-fluid level is present in the gastric fundus.

**Figure 2:**
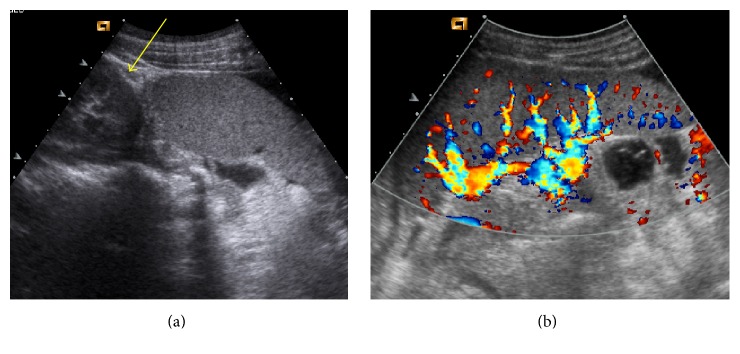
(a) Ultrasonography. The spleen is displaced caudal to the lower pole of the left kidney (yellow arrow). (b) Color Doppler ultrasound examination reveals a turbulent flow in splenic artery secondary to revascularization.

**Figure 3:**
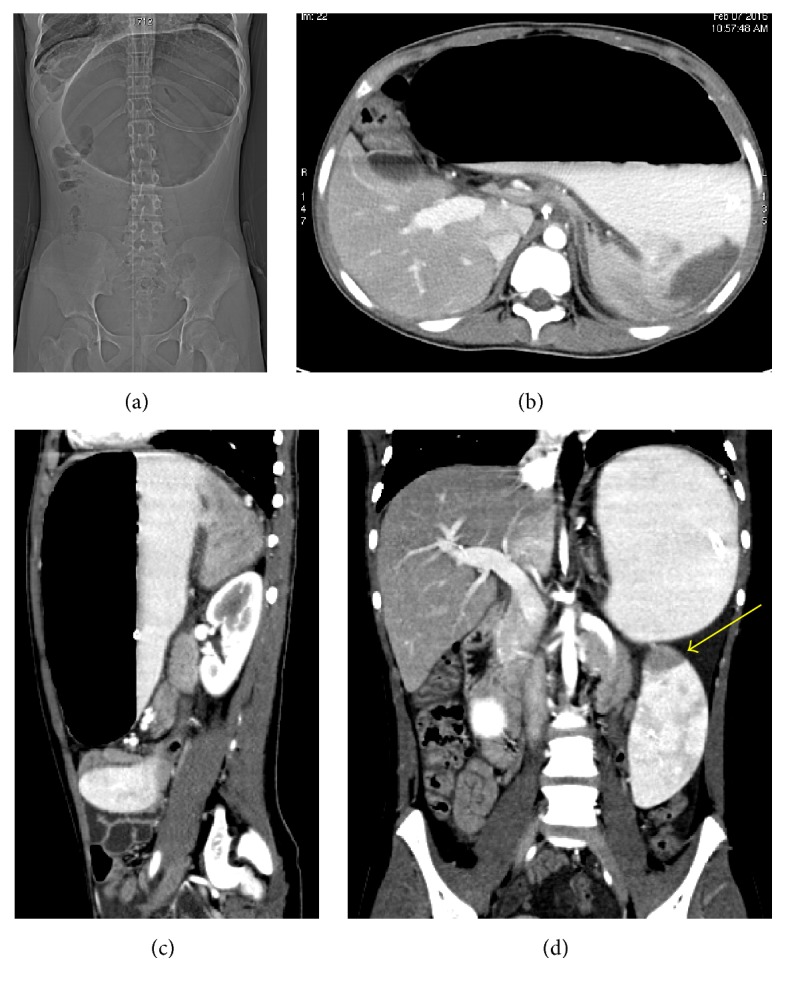
CT scan with oral and intravenous contrast. CT scanogram (a), axial CT scan (b), and sagittal reconstruction (c) show the contrast-filled distended stomach, due to an organoaxial gastric volvulus. The coronal reconstruction (d) reveals a displaced spleen with hypoperfusion of the upper pole (yellow arrow).

**Figure 4:**
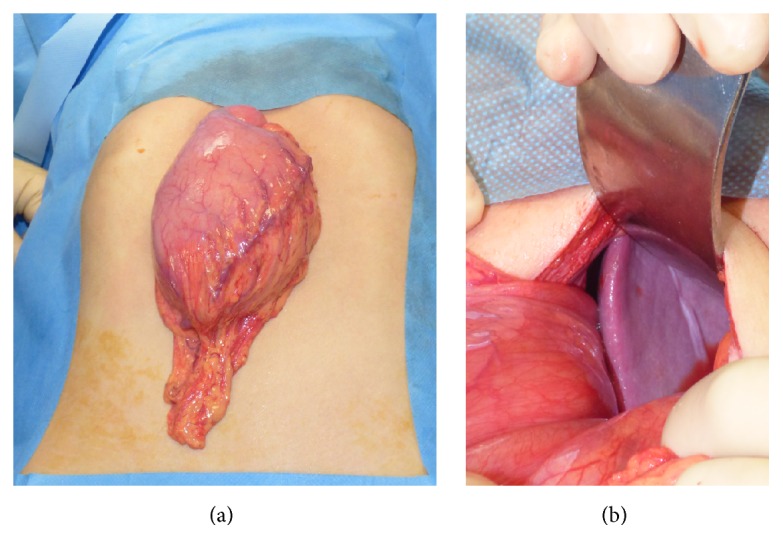
Intraoperative findings: the stomach is rotated with the displacement of the cardia under the pyloric level (a); moreover, the spleen is rotated medially and displaced in the left flank (b).

**Figure 5:**
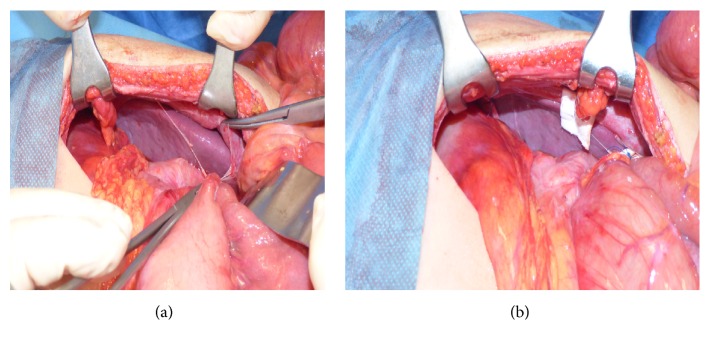
The splenopexy is performed by creation of a retroperitoneal pocket (a) and fixation of the spleen with nonabsorbable sutures mounted on small Teflon pledgets (b).

At admission, a chest and abdominal X-ray was performed finding a severe gastric distension with an air-fluid level and normal diaphragmatic profile (Figure 1). The abdominal ultrasound showed a very mobile spleen displaced lower and medially; at the Doppler evaluation, the spleen vascular flow was present with increased turbulence (Figure 2). These features were corroborated by the oral and intravenous contrast CT scan (Figure 3), which showed a subdiaphragmatic organoaxial gastric volvulus. The esophagogastroduodenoscopy confirmed the diagnosis. With the insight of these findings, an open xifosupraumbilical laparotomy was performed. At surgery, we found a organoaxial gastric volvulus with the displacement of the cardia under the pyloric level (Figure 4(a)); moreover, we noticed a wandering spleen located in the left flank with the absence of short gastric vessels and both gastrosplenic and gastrophrenic ligaments (Figure 4(b)); the hiatal foramen and the diaphragm were normally conformed. There were no signs of ischemia or perforation of both organs. The stomach was derotated, a new anterior Boerema's gastropexy associated with suspension of fundus to the left diaphragm executed, and a splenopexy performed, placing the spleen in a retroperitoneal pocket and fixing it with nonabsorbable sutures mounted on small Teflon pledgets (Figure 5). The postoperative course was uneventful. The patient was dismissed after the 7th day after surgery. The clinical follow-up was uneventful and the upper gastrointestinal study performed after five months showed gastric atony without obstruction.

## 3. Discussion

Wandering spleen is a rare condition characterized by an excessive mobility of the spleen that can “wander” from its anatomical location to any other position in the abdominal cavity, frequently in the lower quadrants due to gravity [1, 11]. Despite being most common in reproductive age women as a result of “acquired” ligamentous laxity, wandering spleen is also described in pediatric age with a peak of incidence in male under the first year [3, 7]. In this group a “congenital” cause has been proposed, secondary to a failure in the development of splenic ligaments, from the dorsal mesentery, that anchor it to the stomach, diaphragm, colon, and retroperitoneum; as a result, the spleen is sustained only by its vascular pedicle with an increased risk of torsion [1, 4–6]. The association with other congenital malformations as prune-belly syndrome, renal agenesis, congenital diaphragmatic hernia and eventration, and gastric volvulus has been previously described [4, 5, 12]; furthermore, a familiar form has been proposed [13].

The incidence is less than 0.25% in splenectomies and probably is underestimated because of an asymptomatic or unspecific clinical presentation [1, 13]; indeed, sometimes the diagnosis is incidental. In the symptomatic cases, the patient can present with an acute abdomen secondary to an acute splenic torsion; in other cases, patients can complain about recurrent abdominal pain with or without evidence of organomegaly (enlarged ectopic or normotopic spleen) secondary to intermittent torsion. Hypersplenism with thrombocytopenia, gastric varicose veins, pancreatitis, and intestinal obstruction are rare complications [4, 11].

The presentation could be very ambiguous in young patients. The diagnostic imaging is essential for the diagnosis and surgery planning. Abdominal Doppler ultrasonography is the first step in the diagnosis of wandering spleen and can be able to demonstrate the abnormal position of the spleen and to detect the presence of pedicle torsion (“whirl” sign). Though, abdominal CT scan might be necessary in some cases, allowing a better anatomical knowledge of the spleen, its vascularity, the presence and the extent of ischemic lesions, and its relationship with the surrounding organs, such as gastric displacement or volvulus [4, 5, 14, 15].

Historically, splenectomy was the treatment of choice for symptomatic wandering spleen; actually it is reserved only when vascular compromise is present. In other cases, splenopexy is preferred with the advantage of preserving the splenic function avoiding the risk of “overwhelming postsplenectomy sepsis” (OPSS). Different techniques have been proposed including suture splenopexy (fixation of the splenic hilum or direct suture of the splenic capsule), colonic displacement, creation of an extraperitoneal pocket, and use of absorbable or synthetic mesh (Dexon, Teflon), with or without gastropexy, using an open or laparoscopic approach [1, 12, 16–18].

Gastric volvulus is a rare but potentially life threatening entity that can occur in both pediatric and adult age. It is defined as an abnormal rotation of more than 180° of the stomach around one of its axes [19]. According to the rotation axis, gastric volvulus is classified into three types: organoaxial, mesenteroaxial, and combined-type. In the organoaxial type, the stomach rotates around its longitudinal axis with an anterior rotation of the greater curvature, which moves from the left to right and from bottom to top. In the mesenteroaxial form, the rotation of the stomach occurs along its transgastric axis, a line joining the middle of both the greater and the lesser curvature; the pylorus or cardia usually rotates anteriorly and the posterior surface of the stomach becomes anterior. In the combined-type, the stomach rotates along both axes [20, 21]. Depending on the etiology, gastric volvulus is classified into primary or idiopathic, caused by a congenital abnormal laxity or absence of the gastric ligaments, and secondary when associated with other defects as gastric malposition or nonfixing, eventration of the diaphragm, diaphragmatic hernia, congenital bands, intestinal malrotation, and wandering spleen, found in 50–75% of cases [19, 20, 22]. In terms of onset, gastric volvulus is classified into acute and chronic. The typical presentation of an acute gastric volvulus is the Borchardt triad: severe epigastric pain and abdominal distension, unproductive retching or vomiting, and difficulty or inability to pass a nasogastric tube into the stomach. In children the clinical presentation is variable and frequently unspecific. In the recurrent form, symptoms involve vague clinical features such as gastroesophageal reflux symptoms, dysphagia, postprandial abdominal pain and distension, vomiting, and chest pain; hematemesis could present in a late stage as a consequence of ischemia [19, 20]. Abdominal X-ray can lead to the diagnosis demonstrating a gastric distension or a gastric double bubble sign, but the oral contrast study is actually the gold standard; CT scans, in particular with radio-opaque contrast, offer a very sensitive diagnostic option and may be useful in clinically unclear cases or in detecting associated anomalies [19, 23]. The insertion of a nasogastric tube or the endoscopic decompression, where possible, can relieve symptoms. Anterior gastropexy with or without phrenofundopexy and/or esophagocardiopexy, fundoplication, and gastrostomy are the surgical options proposed; in case of sever gastric ischemia and necrosis, a gastric resection should be necessary. Laparoscopic and laparotomic techniques are both described [2, 6, 19, 23].

Gastric volvulus and wandering spleen are frequently reported in literature as associated conditions or as additional malformations in patients affected by congenital diaphragmatic hernia. It has been proved that these conditions share a common cause, as previously described [5, 9, 10, 21, 24]. In case of congenital diaphragmatic hernia, these conditions can be the consequence of embryonic components fusion failure due to the intrathoracic organs herniation [6]. Some authors have hypothesized an “acquired” theory for the wandering spleen in which it can be secondary to a disruption of splenic attachments during dissection of splenic flexure in congenital diaphragmatic repair [25].

Although gastric volvulus and wandering spleen are single rare malformations, some authors consider that they are frequently associated. In fact, in an early report, we found 16 of 19 surgically treated cases for gastric volvulus in children had a concurrent wandering spleen [24]. Analyzing the literature, we found a total of 35 cases reported [3, 7, 8, 20, 21, 24, 26–34].

The wandering spleen and left-sided diaphragmatic hernia association was first reported in 1940 [27] and, until today, a total of 13 cases were described [1, 12, 35–40]; all of the reported cases correspond to left-sided diaphragmatic hernia and two cases presented after diaphragmatic hernia repair.

The association between gastric volvulus and diaphragmatic hernia has been frequently reported; according to our data analysis, 38 cases are Morgagni or Morgagni-Larrey hernia, 2 cases are right-sided hernia with sac, and 57 are left-sided hernia, all cases reported during the diaphragmatic hernia repair [2, 6, 19, 22, 23, 32, 41–84]. Among these cases, we determined that the association between gastric volvulus and Morgagni hernia could be considered less frequent than left-sided hernia due to a less probable herniation of the stomach through the anterior diaphragmatic defect.

To our knowledge, there are only three cases that associated all three malformations (gastric volvulus, wandering spleen, and diaphragmatic hernia) [5, 9, 10]. All patients were female. In these patients gastric volvulus and wandering spleen were found during a left-sided diaphragmatic hernia repair, all of which presented with a delayed clinical picture. In two of three cases, a contemporary gastropexy and splenopexy were performed (Table 1).

Some authors propose a conservative management for chronic gastric volvulus with mild or moderate symptoms [19, 23, 41]. Although successful results were obtained with nonsurgical treatment, in our opinion surgery should be considered in order to avoid complications and recurrences [7, 20]. Regarding the technique, a contemporary anterior and fundal gastropexy, as in our case, should be performed, with the aim of fixing the stomach, decreasing the risk of reherniation and/or recurrences and to prevent gastroesophageal reflux by restoring angle of His [2, 6, 20]. We agree with other authors that consider an additional antireflux procedure as excessive, because of spontaneous resolution of gastroesophageal reflux after the gastric volvulus correction [41].

An encoded treatment for wandering spleen is not yet defined. Some authors proposed only a gastropexy in selected cases of wandering spleen with the aim to restore the normal anatomy, preserving the spleen and avoiding the risk related to spleen manipulation [24]. Furthermore, a prophylactic gastropexy is recently recommended in patients with asplenia syndrome due to the increased risk of gastric volvulus [85]; on the basis of this consideration, some authors propose this technique also in patients presenting wandering spleen [3]. Regarding the management in cases associated with gastric volvulus, we believe that a contemporary splenopexy and gastropexy should be performed. In fact, gastropexy alone should not be enough to prevent gastric volvulus relapses, as in our case; moreover, a basic tenet of Boerema gastropexy technique, which consists in the fixation of the lesser curvature of the stomach by the placement of sutures, is the preservation of the greater curvature attachments to the spleen so the fundus will fold back against the anteriorly fixed esophagus [86, 87]. Also the management of incidental finding of wandering spleen is debated; although some authors consider intervention only as an option in case of painful complications [11], we recommend splenopexy, in emergency or elective approach, in order to avoid complications (torsion of both the spleen and the stomach, traumatic injuries, and intestinal obstruction) [1, 35, 36]. Regarding the technique, the fixation of the spleen in a retroperitoneal pocket has the advantage to not injure the capsule and to avoid the splenic vessel kinking [12].

Moreover, in patients who underwent diaphragmatic hernia repair, these malformations should be exclude and, if confirmed, a prophylactic splenopexy and a gastropexy should be performed in the same procedure avoiding the complexity of secondary surgery [35]; in this context, an abdominal approach for diaphragmatic hernia repair allows a contemporary correction of associated malformations [57].

## 4. Conclusion

The association between diaphragmatic hernia, gastric volvulus, and wandering spleen is rarely described, although a wandering spleen and a gastric volvulus might be detected after a diaphragmatic hernia repair, as described in our case. An early diagnosis and treatment are necessary in order to avoid the mentioned complications. According to other authors, we retain that in case of detection of wandering spleen associated with gastric volvulus a prophylactic splenopexy and a gastropexy should be performed in order to avoid complications and relapses.

To our knowledge, this is the first case in which gastric volvulus relapse associated with wandering spleen after a diaphragmatic hernia repair was found; this may be recognized as the first case of wandering spleen after Morgagni hernia repair, considering that all the previously reported cases, also as postoperative finding of diaphragmatic hernia repair, correspond to a left-sided hernia.

## Figures and Tables

**Table 1:** Reported cases of wandering spleen and gastric volvulus associated with diaphragmatic hernia.

Publication	Gender	Age	Type of gastric volvulus	Type of diaphragmatic hernia	Time of presentation related to hernia repair	Comorbidity	Executed technique
Pelizzo et al., 2001 [9]	Female	12 years	Intrathoracic mesenteroaxial gastric volvulus	Left side with sac	Contemporary	Intrathoracic left kidney	Diaphragmatic hernia repair with mesh and sac removal, gastropexy
Liu and Lau, 2007 [5]	Female	44 months	Intrathoracic mesenteroaxial gastric volvulus	Left side	Contemporary	Intrathoracic left kidney	Splenopexy and gastropexy. Repair of diaphragmatic hernia is not described
Aswani et al., 2015 [10]	Female	14 years	Intrathoracic mesenteroaxial gastric volvulus	Left side	Contemporary	Pancreatic volvulus	Splenopexy and gastropexy. Repair of diaphragmatic hernia is not described
Our case, 2016	Female	13 years	Subdiaphragmatic organoaxial gastric volvulus	Previous intervention for Morgagni hernia	After 8 years	None	Splenopexy, anterior gastropexy, and fundophrenicopexy
